# One-step growth of thin film SnS with large grains using MOCVD

**DOI:** 10.1080/14686996.2018.1428478

**Published:** 2018-02-15

**Authors:** Andrew J. Clayton, Cecile M. E. Charbonneau, Wing C. Tsoi, Peter J. Siderfin, Stuart J. C. Irvine

**Affiliations:** ^a^ Centre for Solar Energy Research, OpTIC Centre, College of Engineering, Swansea University, St Asaph, UK; ^b^ Sêr Solar / SPECIFIC, College of Engineering, Swansea University, Swansea, UK

**Keywords:** Thin film SnS, photovoltaics, metal organic chemical vapour deposition, 50 Energy Materials, 209 Solar cell / Photovoltaics, 306 Thin film / Coatings, 500 Characterization

## Abstract

Thin film tin sulphide (SnS) films were produced with grain sizes greater than 1 μm using a one-step metal organic chemical vapour deposition process. Tin–doped indium oxide (ITO) was used as the substrate, having a similar work function to molybdenum typically used as the back contact, but with potential use of its transparency for bifacial illumination. Tetraethyltin and ditertiarybutylsulphide were used as precursors with process temperatures 430–470 °C to promote film growth with large grains. The film stoichiometry was controlled by varying the precursor partial pressure ratios and characterised with energy dispersive X-ray spectroscopy to optimise the SnS composition. X-ray diffraction and Raman spectroscopy were used to determine the phases that were present in the film and revealed that small amounts of ottemannite Sn_2_S_3_ was present when SnS was deposited on to the ITO using optimised growth parameters. Interaction at the SnS/ITO interface to form Sn_2_S_3_ was deduced to have resulted for all growth conditions.

## Graphical abstract

**Figure:**
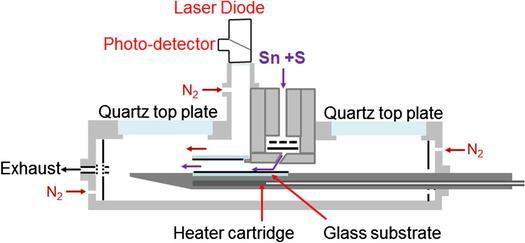

## 1. Introduction

Tin sulphide has been researched for solar cell applications as an absorber layer for many years. However, progress of SnS-based devices has been limited, with only 4.4% certified record [1] conversion efficiency. One contributing factor has been the small grain sizes of SnS [1,2], creating a high density of grain boundary defects that act as carrier recombination centres. Post-growth annealing is generally carried out in either nitrogen, or a sulphur atmosphere such as hydrogen disulphide to compensate for desulphurisation [1]. Control of additional sulphur incorporation can be an issue [3–5] during post-growth annealing treatment in the presence of sulphur leading to the onset of additional undesired phases such as Sn_2_S_3_ and SnS_2_.

Metal organic chemical vapour deposition (MOCVD) offers an alternative one-step process to produce thin film SnS and achieving large grain sizes without the requirement for a post-growth anneal [6]. This simplifies the deposition process to produce SnS films with stoichiometry controlled through the precursor partial pressures during injection into the reaction chamber. Growth of the grains nucleating on the substrate can be expressed through the Johnson–Mehl–Avrami rate equation:

(1) $$\frac{\text{d}\alpha }{\text{d}t}=\left(m+1\right)k\left(1-\alpha \right)\cdot {\left[-ln\left(1-\alpha \right)\right]}^{\frac{m}{m+1}}$$

where *α* is the transformed fraction, *t* is time, *k* is the rate constant and (m + 1) is the Avrami exponent [7]. Nucleation and growth rate are assumed to be independent of time, but the rate constant is related to temperature. Therefore, the rate of grain growth increases at higher temperatures, which can be employed in the MOCVD process whilst having control of the SnS film stoichiometry.

Tin-doped indium oxide (ITO) was used as the substrate as an alternative to molybdenum (Mo). Molybdenum disulphide (MoS_2_) forms at the SnS/Mo interface and can inhibit the photovoltaic (PV) properties of a device if the thickness becomes significant [8,9]. The authors favoured ITO, having a similar work function to Mo [10–13] to give an ohmic back contact, to avoid MoS_2_ formation. As ITO is transparent it also creates the possibility for bifacial illumination, with carriers generated from photons entering the device from the front and back. This report discusses the materials properties of SnS films produced using this approach.

## 2. Experimental details

Tetraethyltin (TET) and ditertiarybutyl-sulphide (DtBS) were used as chemical precursors using a MOCVD process for production of SnS thin films. The carrier gas was hydrogen delivering the precursors directly over the substrate with deposition onto ITO coated boroaluminosilicate glass, with area of 75 × 50 mm^2^ and thickness of 1.1 mm.

Growth temperatures 430–470 °C were used to deposit SnS films. The temperature was controlled using a K-type thermocouple inserted into the graphite susceptor. A N_2_ cooling gas was always employed at the upstream and downstream (exhaust) end sections of the MOCVD chamber.

The TET and DtBS partial pressures ranged from (2.2 to 8.6) × 10^−4^ and (2.2–4.4) × 10^−3^ atm. respectively, which gave S/Sn input ratios of 2.5–20.0. Different S/Sn precursor partial pressure ratios were employed for different temperature regimes focusing on 432 °C and 470 °C set points. Film coverage only became sufficient from ~430 °C. The precursor partial pressure ratio can also be considered as the input precursor concentration ratio, which will be denoted as [S/Sn]^i^, where the suffix ‘i’ represents the concentration ratio injected into the chamber prior to reaction. Total chamber pressure during deposition was equal to 1 atmosphere (atm.). A total flow (F_total_) of 500 standard cubic centimetres per minute (sccm) was used for depositions, which were carried out over 30 or 60 min.

### 2.1. Film characterisation

Scanning electron microscopy (SEM) and energy dispersive X-ray spectroscopy (EDX) were carried out with a Hitachi TM3000 (Hitachi High Technologies, Maidenhead, UK) bench top instrument and used to assess grain size/shape and approximate stoichiometry of the Sn-S deposits. High resolution (HR)-SEM was carried out with a Hitachi S-4800 (Hitachi High Technologies, Maidenhead, UK) instrument equipped with field emission gun (FEG) using beam settings at 2–10 keV and 10 μA. UV/Visible spectroscopy was used with a Varian Cary 5000 (Agilent Technologies, Stockport, UK) to measure the optical properties and X-ray diffraction (XRD) was used with a Phillips X’PERT MPD (PANalytical Ltd., Royston, UK) instrument to determine the thin film crystal structure. Raman spectroscopy measurements were performed with a Renishaw Invia Raman system in backscattering configuration. The laser excitation was 532 nm and a 50x long objective was used (NA: 0.50, spot size ~1 μm). Raman maps were created by obtaining spectra in selected adjacent regions moving from one to another using an X–Y scanning stage (0.6 mW, 5s). Maps were generated by collecting the spectra from all the scanning regions and mapping at a defined intensity for each region.

## 3. Results and discussion

Film deposition with complete substrate coverage was observed on the substrate at temperatures 430–470 °C. The thermal stability of the TET precursor limited growth until process temperatures greater than 410 °C were employed. Higher growth temperatures required greater sulphur partial pressures, with an increase of 3 × 10^**−**4^ atm from 430 to 470 °C, to compensate for its high vapour pressure and desorption from the deposited film under the elevated temperatures.

### 3.1. Grain size

**Figure 1.**
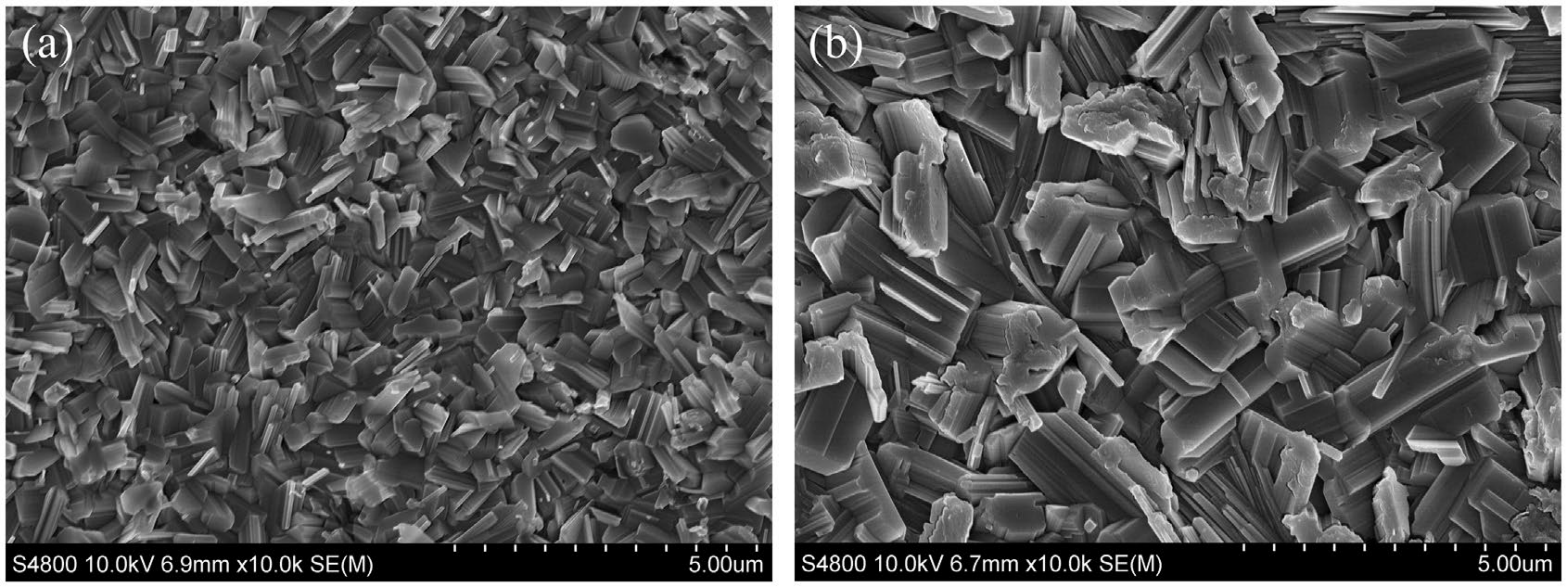
SEM images of film deposited using optimised growth parameters at different growth temperatures: (a) 432 °C; (b) 472 °C.

Grain size was compared using SEM, as shown in Figure 1(a) and (b), of film samples deposited at different temperatures. It was found that higher process temperatures in the range 430–470 °C led to larger grain size as has been reported [14,15] from other studies on SnS. It has also been reported [16] that temperatures of 500 °C and above leads to the decomposition of SnS. A previous study by the authors [6], employing tetramethyltin (TMT) as the Sn source, employed growth temperatures up to 550 °C due to the thermal stability of the Sn precursor. This made it difficult to control the process with likely decomposition of SnS formed at these high temperatures. Grains were still relatively small and not closely packed, resulting in the requirement for post-growth annealing to achieve grain sizes comparable to that observed in this current study. Limited chemical reaction below 500 °C made TMT unsuitable as the Sn source, therefore tetraethyltin (TET) was used in this current study which facilitated SnS film deposition below 500 °C, resulting in large grains for the as-grown films.

The grain enlargement at the higher process temperature [14,15], as shown in Figure 1(b), was significant with approximate grain size increasing from approximately 1 μm in length by 0.5 μm in width at 432 °C to approximately 2 μm in length by 1 μm in width at 470 °C, more than doubling in size. The grain boundary density was therefore much lower for the film produced using the higher temperature conditions during growth. The higher growth temperature will be necessary for producing SnS films with reduced recombination centres which typically reside at grain boundaries, [1,4,15].

The grain sizes were comparable to that for as-grown samples deposited by different groups using thermal evaporation, at a lower substrate temperature of 350 °C [14] and 225 °C [15], respectively. Grain shape differed between the processes with the thermal evaporation produced at 225 °C [15] having a similar shape to the MOCVD study presented here. However, the 225 °C temperature used in the thermal evaporation process also resulted in SnS material desorbing off the surface and SnS films deposited at 200 °C with smaller grains ~0.5 μm could only be used to fabricate devices. Even so, a respectable 2.53% efficient device making use of CdS as n-type buffer was achieved [15].

To realise a further increase in SnS grain size post-growth annealing treatments will have to be considered. This will only be beneficial if the onset of additional Sn_x_S_y_ phases can be prevented.

### 3.2. Composition

**Figure 2.**
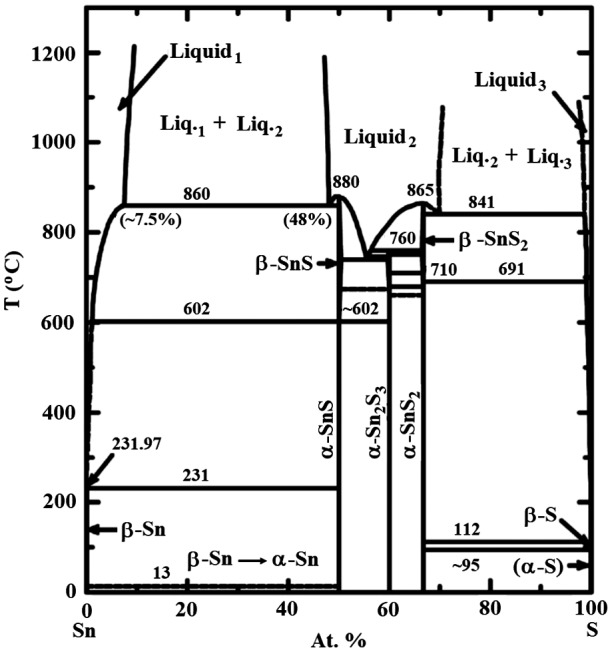
Sn-S phase diagram taken from Ref. [17].

Notes: Reprinted from R.E. Banai, M.W. Horn and J.R.S. Brownson, A review of tin (II) monosulfide and its potential as a photovoltaic absorber, Sol. Energy Mater. Sol. Cells 150 (2016) 112–129, with permission from Elsevier.

One of the advantages that MOCVD has as a process for SnS is the capability to inject the necessary concentrations of sulphur into the films at the higher process temperatures to limit desulphurisation. Desulphurisation is considered a limitation for SnS PV devices [3]. Large grains and SnS phase control will be essential when the SnS absorber is incorporated into a thin film solar cell device. Figure 2 shows the phase diagram for the Sn-S system [17] and indicates that the phase should remain as SnS in the temperature regime employed for experiments if Sn and S incorporation into the film are both at. % equal to 50. This was achieved by varying the [S/Sn]^i^.

EDX was employed post-growth to characterise the S:Sn ratio in the film composition. A wide range of [S/Sn]^i^ was used initially, followed by confinement of the range closer to the point where the film composition changed from Sn-rich to S-rich. This occurred between [S/Sn]^i^ = 5.0 and 7.0, with [S/Sn]^i^ range 5.4–6.2 showing near 1:1 stoichiometry (Table 1). SnS typically has p-type conductivity, with the main contribution from Sn vacancies (V_Sn_) acting as a shallow acceptor [18]. Sn-rich conditions are therefore undesirable for solar cell absorption, which require hole (majority) concentrations to be high. However, large S incorporation leads to Sn_2_S_3_ and SnS_2_ phases, resulting in compensation effects and a change in the Sn oxidation state from +2 to +4 leading to n-type conductivity [19,20]. The Sn_2_S_3_ has mixed Sn oxidation states and the SnS_2_ phase has only the +4 Sn oxidation state. Therefore, near stoichiometric SnS with optimal Sn deficiency is required. In this study, the focus was to achieve single phase SnS films.

**Table 1.** Atomic weight (at. %) composition determined by EDX for films deposited using different S/Sn precursor partial pressure ratios, [S/Sn]^i^.

Sample	T^o^C	DtBS (atm)	TET (atm)	[S/Sn]^i^	S at.%	Sn at.%
TS131	472 ± 1	2.91 × 10^−3^	7.20 × 10^−4^	4.0	43.1	56.9
TS130	472 ± 1	3.26 × 10^−3^	6.56 × 10^−4^	5.0	47.6	52.4
TS117	465 ± 1	3.40 × 10^−3^	6.30 × 10^−4^	5.4	49.7	50.3
TS124	472 ± 1	3.65 × 10^−3^	5.85 × 10^−4^	6.2	50.6	49.3
TS129	473 ± 3	3.83 × 10^−3^	5.50 × 10^−4^	7.0	51.4	48.6
TS118	469 ± 1	4.05 × 10^−3^	5.09 × 10^−4^	8.0	52.5	47.5

### 3.3. Optical properties

All film samples deposited at 470 °C and different [S/Sn]^i^ had strong absorbance over the visible region of the solar spectrum.

The films showed a double absorption edge around 950 and 1100 nm which is indicative of the reported [19–21] band gaps (*E*_g_) of 1.3 and 1.1 eV, respectively, using the relationship:

(2) $$E\;\left(\mathrm{eV}\right)=\;\frac{\mathrm{hc}}{\lambda \times 1.6\times 10^{-19}}$$

**Figure 3.**
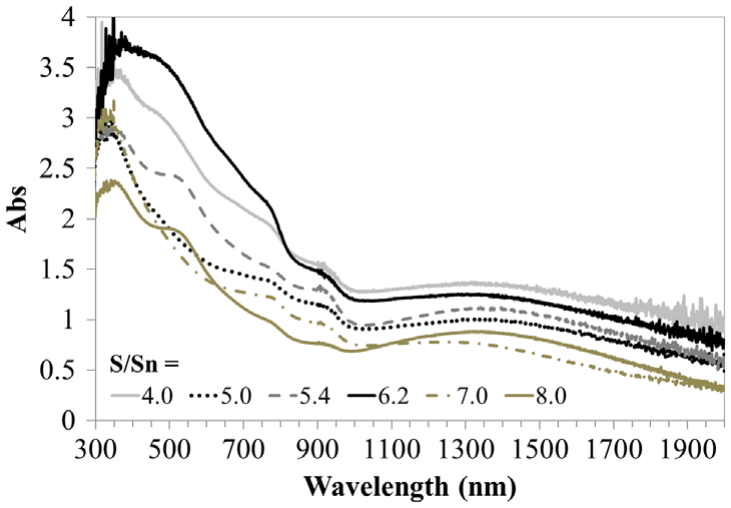
Absorption spectra of films deposited at different [S/Sn]^i^ at a growth temperature of 470 °C.

In Equation (2), *h* is Plank’s constant, *c* is the speed of light, *λ* is the wavelength and 1.6 × 10^−19^ is the equivalent energy in Joules for 1 eV. Film thickness ranged from 1.1 to 1.6 μm for the film samples. A double absorption edge at similar wavelengths was also observed in external quantum efficiency measurements for thin film SnS solar devices [15]. The absorption coefficient (α) was calculated for the film showing the highest absorption in Figure 3, which had [S/Sn]^i^ = 6.2 during growth and resulted with an average 1.6 μm thickness. The α was obtained using the Beer-Lambert relationship:

(3) $$\alpha =\frac{-ln\left(\frac{I}{I_{0}}\right)}{d}=2.303\left(\frac{\mathrm{abs}}{d}\right)$$

where *I* is the light intensity detected through the film sample and *I*_*0*_ is the light intensity detected through the spectrometer without the sample present. The absorption is denoted by *abs* and the thickness is represented by *d*. Over the visible range of the solar spectrum the *α* was calculated to be (3.4–5.2) × 10^4^ cm^−1^.

**Figure 4.**
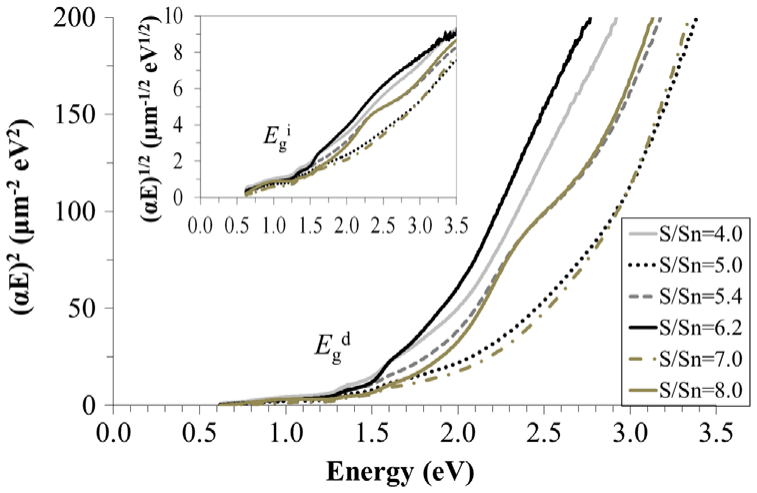
Band gap calculation of films deposited at different [S/Sn]^i^ using absorption coefficient × energy squared versus energy, (αE)^2^ vs. E, for direct ($E_{g}^{d}$) transitions and the square route of absorption coefficient × energy versus energy, (*αE*)^1/2^ vs. E, for indirect ($E_{g}^{i}$) transitions (inset).

SnS has both a direct and indirect *E*g_._ Tauc plots (Figure 4 were calculated for both using (*αE*)^2^ vs. *E* and (*αE*)^1/2^ vs. *E* for direct $E_{g}^{d}$ and indirect $E_{g}^{i}$, respectively. The plots were consistent with that reported for SnS [19–21]).

### 3.4. Material phase

**Figure 5.**
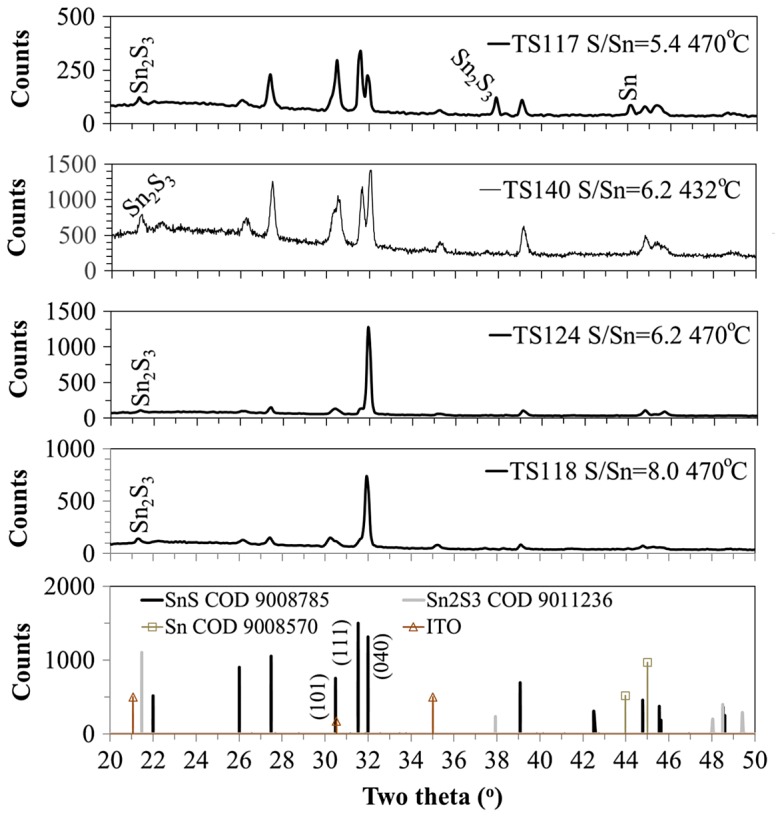
XRD of films deposited at 470 °C using different [S/Sn]^i^ equal to 5.4, 6.2 and 8.0, which showed cross-over of SnS 1:1 stoichiometry determined from EDX measurements.

Note: A film deposited at 432 °C and [S/Sn]^i^ equal to 6.2 is included for comparison.

XRD was used to determine the phases present in the films relative to the [S/Sn]^i^. SnS is typically orthorhombic and has many peaks associated with its lattice structure between two theta (2*θ*) angles 20^o^–70^o^. Reference diffraction peaks for different materials phases were obtained from the Crystallography Open Database (COD) and are compared with the diffraction patterns obtained for different [S/Sn]^i^ in Figure 5. The diffraction peaks associated with other phases, such as metallic tin and ottennamite Sn_2_S_3_, were observed to occur below a 2*θ* angle of 50^o^. The main phase impurity to be observed was Sn_2_S_3_. Figure 5 shows the XRD patterns for different samples deposited at 470 °C using different [S/Sn]^i^ and also includes the diffractogram for an equivalent SnS film deposited with [S/Sn]^i^ = 6.2 and growth temperature of 432 °C.

The film produced with [S/Sn]^i^ = 5.4 shows a diffraction peak representing the presence of Sn. EDX showed (Table 1) a near 1:1 stoichiometry, but Figure 5 shows this not to be the case with other phases being detected. To obtain SnS with Sn oxidation state of +2 the Sn in the TET needs to be reduced from the oxidation state of +4. This most likely occurs by interaction with the hydrogen carrier gas at the elevated temperatures.

The two films deposited at 470 °C with [S/Sn]^i^ of 6.2 and 8.0 showed similar XRD diffraction peaks. The main peak for these two samples at 2*θ* = 31.9^o^, corresponding to the (040) plane in SnS and becomes more dominant for the film with [S/Sn]^i^ = 6.2. EDX for this film (Table 1) showed the closest 1:1 stoichiometry for SnS, with the film produced at [S/Sn]^i^ = 8.0 showing S-rich conditions. There was no evidence in the XRD of the SnS_2_ phase being present in the films using the S-rich conditions as characterised by EDX. XRD was not carried out on films having been produced with the highest [S/Sn]^i^, where the SnS_2_ phase would most likely have been observed. It has been reported [22] that as the sulphur content increases the SnS film self-compensates as the number of Sn vacancies increase, with the Sn oxidation state changing from +2 to +4. This would lead to higher probability of the Sn_2_S_3_ phase impurity forming, with mixed oxidation states for Sn. All films had a small diffraction peak at 2*θ* = 21.4^o^, including the film deposited with the smallest S/Sn = 5.4. It is likely that this peak is associated with Sn_2_S_3_ (COD9011236), but it is similar to a 2*θ* diffraction angle assigned to the ITO substrate [23–25].

Di Mare et al. reported [3] the issue of overlapping XRD peaks between SnS and Sn_2_S_3_. The phase diagram in Figure 2 suggests that the Sn_2_S_3_ phase should not be detected for Sn-rich films. With excess Sn, incomplete reduction is possible, with Sn +4 material being incorporated from the TET precursor into the film. According to the EDX characterisation (Table 1) the film with [S/Sn]^i^ = 5.4 should be slightly Sn-rich. It could be that this is the reason for the discrepancy in the XRD (Figure 5) showing detected Sn_2_S_3_ in this sample, the phase having mixed Sn oxidation states.

The diffractogram of the sample deposited at lower temperature (432 °C) and [S/Sn]^i^ = 6.2 showed more intense peaks for other reflection angles compared to the equivalent sample deposited at 470 °C, which had a preferred orientation along the 0,4,0 plane. There was a more prominent peak for Sn_2_S_3_ at the 21.4^o^ angle revealing a stronger presence of this phase. This correlates with other work [15] where lower growth temperatures led to greater detection of the Sn_2_S_3_ phase. As sulphur has a high vapour pressure, elevated temperatures are likely to evaporate it from the growing film, whereas at lower temperatures more sulphur will be incorporated. This is the reason why greater sulphur partial pressures are required at the higher growth temperatures for preserving the SnS stoichiometry.

**Figure 6.**
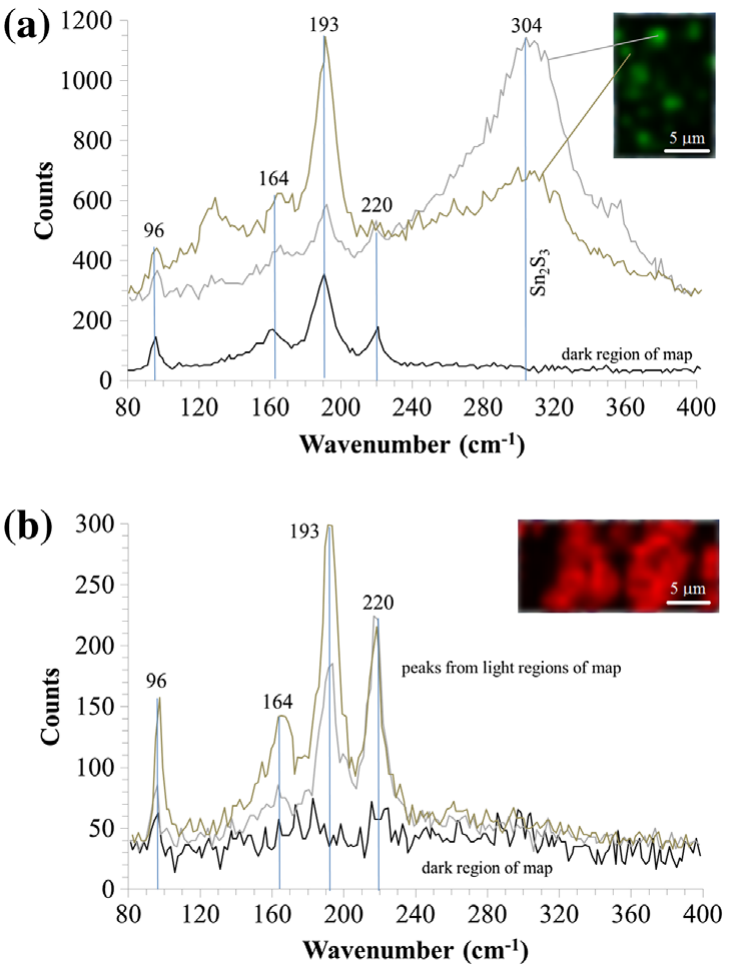
Raman spectroscopy of different film regions for SnS samples deposited at optimised growth parameters on (a) ITO mapped at a 304 cm^−1^ frequency and (b) Mo mapped at a 193 cm^−1^ frequency.

Raman spectroscopy was carried out on the film with [S/Sn]^i^ = 6.2 to confirm if there was any Sn_2_S_3_ phase present (Figure 6). According to Di Mare et al. [3], vibrational frequencies for SnS occurred below 300 cm^−1^, with a main shift at 193 cm^−1^. Shifts associated to the Sn_2_S_3_ phase occurred at 304 cm^−1^ and for the SnS_2_ phase at 312 cm^−1^. Different film sample regions were mapped at vibrational frequencies of 193 cm^−1^ and 304 cm^−1^, the main peaks associated to SnS and Sn_2_S_3_, respectively. Figure 6(a) shows the Raman shifts associated with the SnS film deposited onto ITO. For comparison, Raman spectroscopy was carried out on an equivalent film deposited onto Mo, mapping different regions of the film sample at the same two frequencies, showing the Raman map at 193 cm^−1^ in Figure 6(b).

The film on ITO showed correlation to SnS mapping at the frequency of 193 cm^-1^, but showed inhomogeneous phase distribution when mapped at 304 cm^−1^, as shown in Figure 6(a), indicating a non-uniform presence of Sn_2_S_3_ across the characterised sample area. In some cases, no Sn_2_S_3_ was detected when mapping at 304 cm^−1^, with only SnS being present. However, the intensity of the Raman shift at 304 cm^−1^ was in some cases very high, signifying a strong presence of the Sn_2_S_3_ phase. The presence of the Sn_2_S_3_ phase in the film could be due to:

• Use of a Sn+4 precursor (TET) with incomplete Sn reduction during film growth.
• Interaction of the growing SnS film at the substrate interface through reaction with the Sn from the ITO layer.

The film deposited onto Mo using the same growth parameters, represented in Figure 6(b) with Raman mapping at a frequency of 193 cm^−1^, shows a strong correlation to SnS. These SnS peaks are in close agreement with Raman maps of SnS reported by other groups [3,4]. The equivalent map at 304 cm^−1^ did not show any correlation in any sample region confirming no detection of the Sn_2_S_3_ phase. The film was therefore single phase SnS. The authors believe that the presence of Sn_2_S_3_ in the film deposited onto ITO was most likely be due to the high deposition temperatures facilitating the interaction of the growing Sn-S film with Sn at the SnS/ITO interface. This may follow a similar mechanism to the formation of MoS_2_ at the SnS/Mo interface.

## 4. Conclusions

The MOCVD process was successfully used to produce SnS films with large grains >1 μm without the requirement for a post-growth annealing step. The as-grown films were highly absorbing with absorption coefficients ranging (3.4–5.2) × 10^4^ cm^−1^ in the visible range of the solar spectrum, showing $E_{g}^{d}$ ~ 1.3 eV and $E_{g}^{i}$~ 1.1 eV, in agreement with literature reports. EDX showed a film deposited with [S/Sn]^i^ = 6.2 having the closest 1:1 stoichiometry for single phase SnS, which also showed the closest correlation to the optical properties reported in the literature.

XRD revealed the presence of the Sn_2_S_3_ phase for all films deposited on to ITO, including the sample with Sn-rich composition according to EDX, which was surprising. Raman spectroscopy identified that the Sn_2_S_3_ phase was indeed present for the optimised SnS film on ITO. Comparison was made to an equivalent film on Mo which showed no Raman shifts associated to the Sn_2_S_3_ phase, with only detection of shifts associated to single phase SnS. It is likely that the Sn from the ITO interacts with the growing SnS at the high deposition temperatures to form Sn_2_S_3_. This small amount of Sn_2_S_3_, if residing at the SnS/ITO interface, may not necessarily be detrimental to solar cell performance if the thickness is minimal, behaving in a similar way to MoS_2_ at the SnS/Mo interface.

## Disclosure statement

No potential conflict of interest was reported by the authors.

## Funding

This work was supported by the Welsh European Funding Office from the European Regional Development Fund.
